# Estimating the size of fields in biomedical sciences

**DOI:** 10.1128/msystems.00652-23

**Published:** 2023-12-06

**Authors:** Quigly Dragotakes, Arturo Casadevall

**Affiliations:** 1 Department of Molecular Microbiology and Immunology, Johns Hopkins Bloomberg School of Public Health, Baltimore, Maryland, USA; University of California San Diego, La Jolla, California, USA

**Keywords:** research fields, size of fields, bibliometric, organization

## Abstract

Scientific research output has increased exponentially over the past few decades, but not equally across all fields of study, and we lack clear methods for estimating the size of any given field of research. Understanding how fields grow, change, and are organized is essential to understanding how human resources are allocated to the investigation of scientific problems. In this study, we estimated the size of certain biomedical fields from the number of unique author names appearing in field-relevant publications in the PubMed database. Focusing on microbiology, where the size of fields is often associated with those who work on a particular microbe, we find large differences in the size of its subfields. We found that plotting the number of unique investigators as a function of time can show changes consistent with growing or shrinking fields. In general, the number of unique author names associated with a particular microbe correlated with the number of disease cases attributed to that microbe, suggesting that the microbiology field workforce is deployed in a manner consistent with the medical importance of the microbe in question. We propose that unique author counts can be used to measure the size of the workforce in any given field, analyze the overlap of the workforce between fields, and compare how the workforce correlates to available research funds and the public health burden of a field.

**IMPORTANCE:**

Science and its individual fields are growing at spectacular rates along with the number of papers being generated each year. However, we lack methods to investigate the size of these fields, many times relying on anecdotal knowledge on which fields are “hot topics” or oversaturated. Thus, we developed a bibliometric method analyzing authorship information from PubMed to estimate the size of fields based on unique author counts. Our major findings are that unique author counts serve as an efficient measurement of the size of a given field. Additionally, the size of a biomedical science field correlates to its public health burden when compared to case numbers. This method allows us to compare growth rates, workforce distribution, and the allocation of resources between fields to understand how scientific fields self-regulate. These insights can, in turn, help guide policymaking, for example, in funding allocation, to ensure fields are not neglected.

## INTRODUCTION

As the knowledge domain of science increased, there was a need for specialization (1). Early scientists were generalists who could be expected to have some knowledge of the totality of human epistemic endeavors and were known as natural philosophers. For example, Newton created calculus, formulated the laws of Newtonian physics, knew astronomy, was interested in theology, and may have spent much of his time working on alchemy, a precursor discipline to chemistry. However, by the 19th century, the knowledge base had grown so large that scientists specialized in the different areas of the natural sciences such as physics, chemistry, and biology. These, in turn, underwent further specialization in the 20th century into discrete fields that focused on specific problems.

Today, science is organized into fields, and many fields have several subfields. We will use the fields of microbiology and immunology as examples, as these are the ones we are most familiar with. Both microbiology and immunology are subfields of the larger field of biomedical sciences, which is, in turn, a subfield of biology (1, 2). Hence, fields constitute subgroupings of scientists working on discrete problems often within fussy epistemic boundaries. Fields are also the sociological units by which science is organized since they constitute a major source of friends and personal contacts for scientists (3). Herrera et al. used network analysis to map the connectivity and size among fields within physics (3). Chavalarias and Cointet developed a method to infer phylomemetic patterns from the published literature and used its density to ascertain the growth and decline of fields (4). The question of field size is important for understanding how human resources are invested in areas of science and could be significant for scientific progress given that large fields may stymie the development of new ideas and concepts (5).

Despite the importance of field organization to science, we could find little or no information on the size of fields. Although scientists anecdotally know that some fields are larger than others, remarkably little has been done to quantitate the size of fields. This is an important problem because the size of a field is a measure of how much human capital is devoted to a particular problem. For example, when the COVID-19 pandemic began in 2019, it may have been useful to know the number of scientists with experience in coronavirus or viral vaccines as this would have provided a measure of human resources initially available to confront such a threat. Knowing the size of fields is also important when considering the efficient allocation of scarce resources. However, estimating the size of fields is not an easy task. Scientists move between fields and often engage in cross-field research, creating fuzzy boundaries that defy easy categorization. Assuming one can identify a measure for field size, there are other obstacles to accurate enumeration. For example, many scientists who published in this journal work on various problems simultaneously and, therefore, may belong to more than one field at any given time. In this regard, the work of these authors could fit within the fields of immunology or microbiology, or their interface, depending on how their contributions are assessed. Second, fields evolve with time with some increasing in size and others shrinking. In this study, we approach the problem of the size of fields using bibliometric approaches whereby the number of scientists with a given name is associated with a subject and have developed software that allows one to estimate the size of fields. We use microbiology to explore this topic since this is a subfield of biomedical sciences that is sectioned by the microbes studied (2).

We hypothesize that the size of a given field can be estimated by counting the total unique authors in published articles of the said field. In this study, we estimated the size of subfields within the field of microbiology by counting the number of unique names identified with specific microbes and found a large variation. We anticipate that the number of papers associated with a topic is also a measure of the size of a field but predict that counting authors rather than papers will provide a better estimate of field size since each individual is unique, and fields are composed of people, not papers. For example, a publication describing a microbe interacting with a macrophage could belong to the fields of microbiology, immunology, and cell biology while an author in the paper is a unique entity who is potentially traceable by name or ORCID. Furthermore, focusing on individuals mitigates confounders arising from differences in laboratory productivity. For example, a field composed of 100 laboratories that each produces one paper per year is larger than a field composed of one laboratory that produces 100 papers per year, but this distinction could not be made by only counting the research output.

## MATERIALS AND METHODS

A given field was denoted by a search term, which was used to query the PubMed database (https://pubmed.ncbi.nlm.nih.gov/). Basic PubMed searches are used in which the query is matched to the title, abstract, and keywords of articles. All found publications were then downloaded using Entrez Direct, and a list of all authors was taken from the recorded author bylines (6). Unique names were determined by the first and last names documented in the PubMed database. Data were only collected for articles with a publication date and authors with a recorded first and last name entry. Funding information was collected from NIH RePORTER (https://reporter.nih.gov/) with search terms matching those of PubMed queries. Case burden information was gathered from literature and CDC national reporting (7, 8). Author information was parsed and analyzed in R (version 4.3.0) (9).

## RESULTS

### Estimating field size by counting unique authors

**Fig 1:**
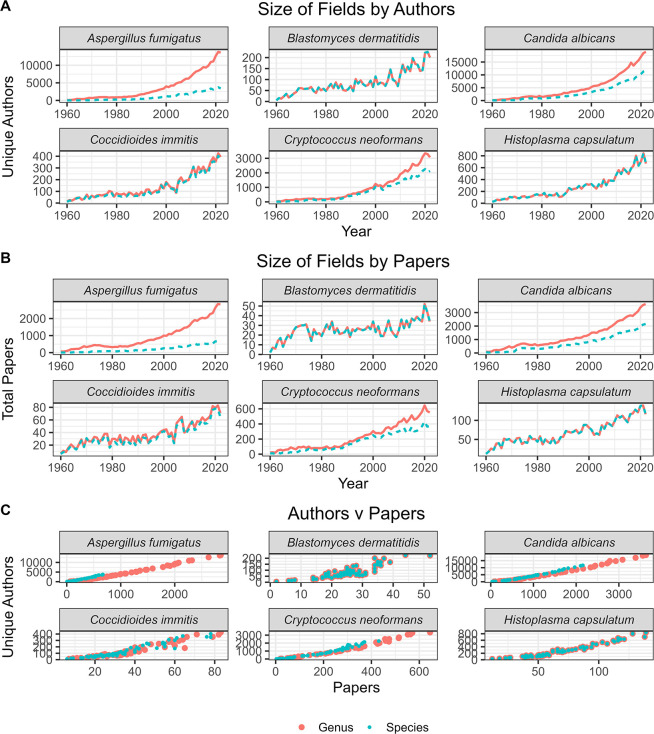
Estimating the size of fields by author count. (A) The size of six significant fungal fields was calculated by searching PubMed for either just the genus (red) name or the genus and species (blue) name. (B) The size of these same six fields calculated by total papers per year. (C) The number of unique authors in each field each year was compared to the total number of publications. An overall positive correlation was observed but varied by field. Species designations are as follows: *Aspergillus fumigatus, Blastomyces dermatitidis, Candida albicans, Coccidioides immitis, Cryptococcus neoformans,* and *Histoplasma capsulatum*.

**Fig 2:**
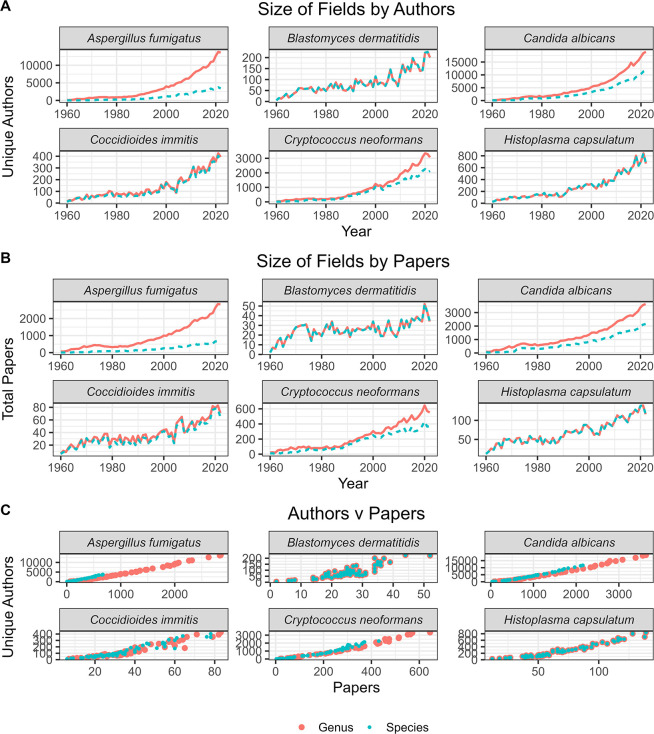
Size of field comparison between author and paper count. We compared paper count to unique author count as estimates for the size of fields. Overall, the two correlate well, suggesting that the paper could be used as a broad surrogate measure of field size.

We first estimated the size of given fields by counting all unique authors that appear in PubMed-deposited articles. We found that each of these fields had a general upward trend in the number of unique authors over time and that individual species searches heavily resembled that of the total genus searches (Fig. 1A; Tables 1 and 3). We next measured field size by the total number of papers published within a given field each year and found similar overall trends (Fig. 1B). To investigate how closely the number of unique authors in a field correlated to the number of papers published in any given year, we directly compared unique authors and total papers. We found that there is a strong overall correlation (0.98 via Pearson and Spearman) between authors and publications, with the strength of the correlation in individual fields showing more variation (Fig. 1C and 2). While the number of unique authors in a field is clearly reliant on publications being generated in the said field, we believe using author counts can generate a deeper image of a field’s growth. Counting authors accounts for trainees, collaborations, etc., which would otherwise be missed by just counting the publication itself.

**TABLE 1:** Size of fields in 2022 as indicated by unique author names

Field	Size in 2022^*a*^
*Aspergillus fumigatus*	3,406 (13,602)
*Blastomyces dermatitidis*	200 (200)
*Candida albicans*	11,681 (18,949)
*Coccidioides immitis*	369 (394)
*Cryptococcus neoformans*	2,128 (3,070)
*Histoplasma capsulatum*	669 (669)
*Paracoccidioides*	242
*Pneumocystis jirovecii*	1,307
Mucorales	1,536
*Candida auris*	1,550
*Fusarium*	8,304
*Candida parapsilosis*	1,823
*Candida tropicalis*	1,637
*Scedosporium*	452
*Cryptococcus gattii*	539
*Penicillium marneffei*	357
*Lomentospora prolificans*	461
*Candida glabrata*	2,210
*Candida krusei*	915
Mycetoma	417
*Caenorhabditis elegans*	7,903
*Saccharomyces cerevisiae*	15,014
*Drosophila melanogaster*	8,296
*Danio rerio*	18,564
Poliovirus	1,877
Variola major	2,050
Phage	14,243
SV40	614
ZFNs	230
TALENs	900
CRISPR	35,642
HIV	77,779
Tuberculosis	43,857
Malaria	25,303
Aquaporin	5,676
Prion	5,067
Coronavirus	272,352
Influenza	39,554
Salmonellosis	6,384
Zika	6,982
VHF	9,986
Cholera	3,951
Dengue	11,362

^*a*^ Calculated sizes of fields in the latest full year at the time of publication. Parentheses denote genus-only search term.

To ensure that our workflow accurately captured a snapshot of a field, we compared the known literary corpus of three individual authors. As expected, we found that the Entrez Direct PubMed search was a reliable method for returning relevant articles (Table 2). We also noted that query formation is an essential step in the analysis as even slight differences in a query can drastically alter the list of returned articles, especially when dealing with larger fields (Fig. S6B). Finally, we investigated fitting both linear and exponential growth models to each of the various analyzed fields and found that exponential growth better explains almost all of them (Fig. S1). There are clear exceptions to this though, such as the simian vacuolating virus 40 (SV40 field, which experiences both growth and decline with time.

**TABLE 2:** Manual and automatic testing of PubMed searches

Investigator^*a*^	Manual assignment	Automatic assignment	Total
1	12	12	16
2	19	19	29
3	13	13	21

^*a*^ We analyzed the research corpus of three individual fungal researchers comparing manual or automatic assignment of these papers to a given topic: *Cryptococcus neoformans*. In each sample, manual assignment and automatic assignment of these papers fully agreed. For example, investigator 1 has 16 total published articles. We manually attributed 12 of those papers to the cryptococcal field, and the automated search attributed the same 12 papers to that field. In each case, human and automatic associations agreed.

### Field size correlates to disease burden

**Fig 3:**
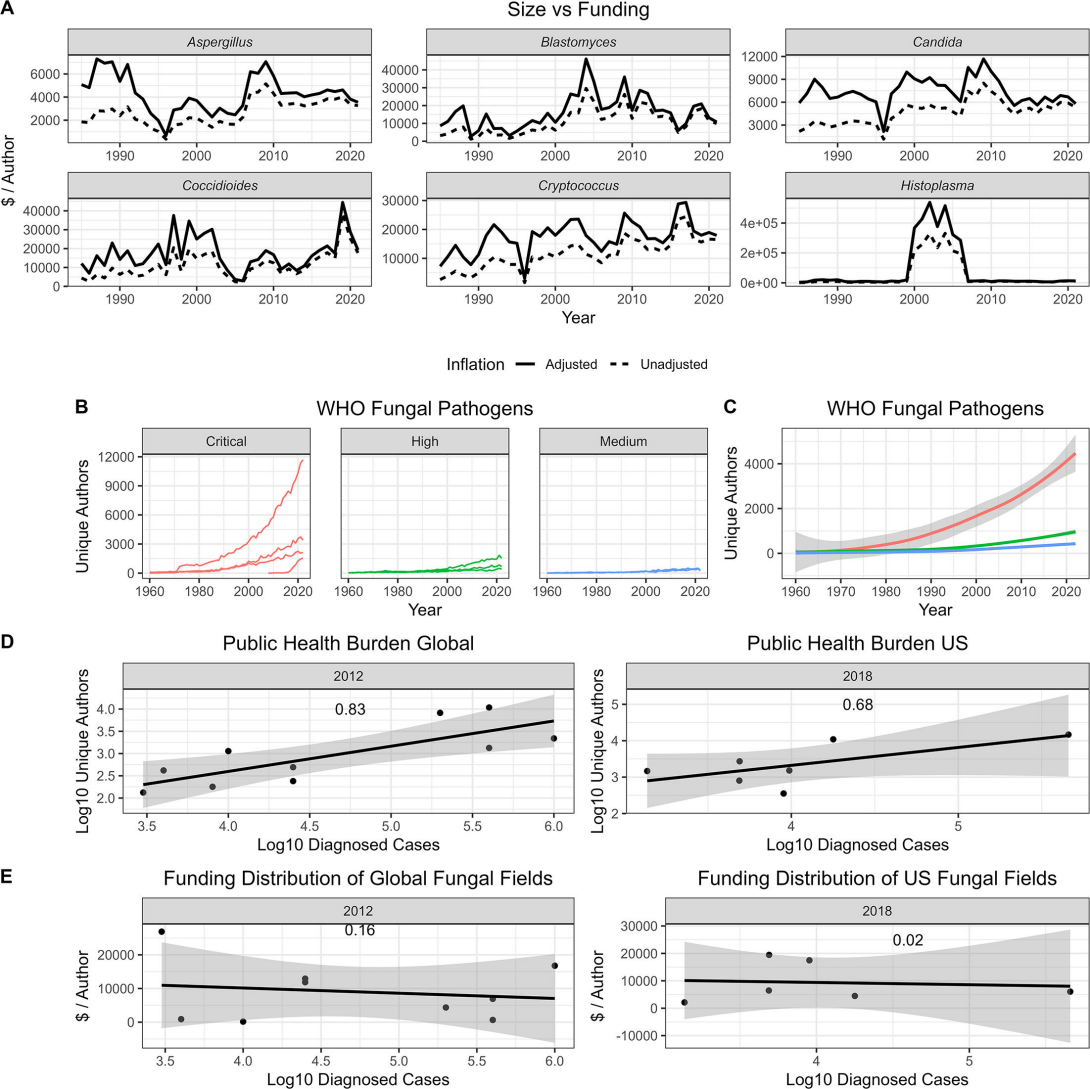
NIH funding and health burden according to field size. (A) We report the number of US dollars ($) awarded to a field compared to the total authors within that field over the course of all available NIH RePORTER data and adjusted for inflation according to the average annual consumer price index (CPI) for all urban consumers. (B and C) Size of fields compared to global health burden of pathogen. We determined the size of fields for every fungal pathogens recently categorized by the WHO in terms of criticality. Critical and high-priority fungal fields are larger than medium, but all categories are growing steadily in size. (B) A combined regression for unique author analysis of all fungal diseases of a given category, pooled together. (C) Individual author analyses for each fungal pathogen within a category. (D) Workforce compared to public health burden of given diseases. In both global and US-specific diseases, the size of a field correlates to disease burden. (E) Funding per author compared to diagnosed case burden of various fungal diseases. The numbers in panels D and E depict Spearman correlation coefficients.

The number of cases attributed to a particular disease is a measure of the importance of that disease to society. Knowing the size of the fields working on specific diseases is a measure of the resources invested in studying a disease, which provides a mechanism for evaluating the consistency of resource allocations. We first investigated whether the total amount of funding for a given pathogen correlated to the size of a given field. We found that, generally, the increase in funding outpaced the increase in authors for all six of our fungal fields, resulting in higher dollar per author funding for the fields (Fig. 3A; Table 3) over time. This trend remained largely the same after adjusting for inflation. Histoplasma represented an exception to this trend, seeing a large spike in funding after the year 1999. There was no sudden outbreak of histoplasmosis at this time; however, the NIH budget was roughly doubled between 1998 and 2003 before flattening again and may account for this sudden burst in Histoplasma funding (10, 11).

**TABLE 3:** Raw data of case burden, funding, and field size for the fields analyzed in this manuscript

Year	Sample name	Unique	Papers	Funding	Query	Inflation
1985	*Cryptococcus*	287	104	761372	Cryptococcus	Unadjusted
1986	*Cryptococcus*	281	96	1111486	Cryptococcus	Unadjusted
1987	*Cryptococcus*	320	95	1792586	Cryptococcus	Unadjusted
1988	*Cryptococcus*	369	111	1551127	Cryptococcus	Unadjusted
1989	*Cryptococcus*	488	141	1607585	Cryptococcus	Unadjusted
1990	*Cryptococcus*	494	142	2468405	Cryptococcus	Unadjusted
1991	*Cryptococcus*	580	174	4809996	Cryptococcus	Unadjusted
1992	*Cryptococcus*	572	168	5903017	Cryptococcus	Unadjusted
1993	*Cryptococcus*	650	187	6072378	Cryptococcus	Unadjusted
1994	*Cryptococcus*	674	208	5321315	Cryptococcus	Unadjusted
1995	*Cryptococcus*	747	218	5889374	Cryptococcus	Unadjusted
1996	*Cryptococcus*	825	229	1331554	Cryptococcus	Unadjusted
1997	*Cryptococcus*	891	253	9354782	Cryptococcus	Unadjusted
1998	*Cryptococcus*	954	257	9294949	Cryptococcus	Unadjusted
1999	*Cryptococcus*	979	268	11407984	Cryptococcus	Unadjusted
2000	*Cryptococcus*	1,237	305	13003499	Cryptococcus	Unadjusted
2001	*Cryptococcus*	1,108	278	13699384	Cryptococcus	Unadjusted
2002	*Cryptococcus*	1,142	279	16352078	Cryptococcus	Unadjusted
2003	*Cryptococcus*	1,025	274	15098658	Cryptococcus	Unadjusted
2004	*Cryptococcus*	1,201	298	13778080	Cryptococcus	Unadjusted
2005	*Cryptococcus*	1,345	346	13818571	Cryptococcus	Unadjusted
2006	*Cryptococcus*	1,411	357	11945706	Cryptococcus	Unadjusted
2007	*Cryptococcus*	1,411	331	15672042	Cryptococcus	Unadjusted
2008	*Cryptococcus*	1,544	362	16803764	Cryptococcus	Unadjusted
2009	*Cryptococcus*	1,608	371	30036933	Cryptococcus	Unadjusted
2010	*Cryptococcus*	1,914	426	32154570	Cryptococcus	Unadjusted
2011	*Cryptococcus*	2,010	434	32080371	Cryptococcus	Unadjusted
2012	*Cryptococcus*	2,185	473	28556554	Cryptococcus	Unadjusted
2013	*Cryptococcus*	2,092	420	27894362	Cryptococcus	Unadjusted
2014	*Cryptococcus*	2,280	460	28144107	Cryptococcus	Unadjusted
2015	*Cryptococcus*	2,621	514	38248824	Cryptococcus	Unadjusted
2016	*Cryptococcus*	2,510	457	58999309	Cryptococcus	Unadjusted
2017	*Cryptococcus*	2,509	474	61322799	Cryptococcus	Unadjusted
2018	*Cryptococcus*	2,719	515	45127321	Cryptococcus	Unadjusted
2019	*Cryptococcus*	3,096	563	48439212	Cryptococcus	Unadjusted
2020	*Cryptococcus*	3,344	645	55699604	Cryptococcus	Unadjusted
2021	*Cryptococcus*	3,290	571	54339891	Cryptococcus	Unadjusted
2022	*Cryptococcus*	3,070	552	37044435	Cryptococcus	Unadjusted
1985	*Candida*	2,197	728	4740488	Candida	Unadjusted
1986	*Candida*	2,126	712	5638683	Candida	Unadjusted
1987	*Candida*	2,308	748	8029749	Candida	Unadjusted
1988	*Candida*	2,499	789	7993649	Candida	Unadjusted
1989	*Candida*	3,023	911	8271536	Candida	Unadjusted
1990	*Candida*	2,891	919	8550219	Candida	Unadjusted
1991	*Candida*	3,133	951	10354522	Candida	Unadjusted
1992	*Candida*	3,505	1,014	12404071	Candida	Unadjusted
1993	*Candida*	3,512	1,008	12140878	Candida	Unadjusted
1994	*Candida*	3,620	1,034	11712181	Candida	Unadjusted
1995	*Candida*	3,896	1,128	12193840	Candida	Unadjusted
1996	*Candida*	4,198	1,121	4818405	Candida	Unadjusted
1997	*Candida*	4,221	1,138	16335508	Candida	Unadjusted
1998	*Candida*	4,545	1,245	20333765	Candida	Unadjusted
1999	*Candida*	4,674	1,269	26331886	Candida	Unadjusted
2000	*Candida*	5,269	1,358	27965059	Candida	Unadjusted
2001	*Candida*	5,691	1,506	29501983	Candida	Unadjusted
2002	*Candida*	5,781	1,522	32598677	Candida	Unadjusted
2003	*Candida*	5,973	1,494	30718254	Candida	Unadjusted
2004	*Candida*	6,570	1,626	34542213	Candida	Unadjusted
2005	*Candida*	6,687	1,674	31723894	Candida	Unadjusted
2006	*Candida*	7,071	1,748	29406926	Candida	Unadjusted
2007	*Candida*	7,587	1,747	56391592	Candida	Unadjusted
2008	*Candida*	8,022	1,902	54509045	Candida	Unadjusted
2009	*Candida*	8,566	2,010	72859705	Candida	Unadjusted
2010	*Candida*	9,283	2,163	68944158	Candida	Unadjusted
2011	*Candida*	10,185	2,337	68198511	Candida	Unadjusted
2012	*Candida*	10,810	2,420	58286036	Candida	Unadjusted
2013	*Candida*	11,394	2,446	50131399	Candida	Unadjusted
2014	*Candida*	12,195	2,600	61044131	Candida	Unadjusted
2015	*Candida*	13,047	2,726	68121434	Candida	Unadjusted
2016	*Candida*	14,764	2,789	67618941	Candida	Unadjusted
2017	*Candida*	13,627	2,753	75721306	Candida	Unadjusted
2018	*Candida*	14,720	2,927	76034711	Candida	Unadjusted
2019	*Candida*	15,651	3,085	93522398	Candida	Unadjusted
2020	*Candida*	17,068	3,391	100541073	Candida	Unadjusted
2021	*Candida*	18,517	3,560	99032829	Candida	Unadjusted
2022	*Candida*	18,949	3,633	76864438	Candida	Unadjusted
1985	*Histoplasma*	134	40	189181	Histoplasma	Unadjusted
1986	*Histoplasma*	170	54	373305	Histoplasma	Unadjusted
1987	*Histoplasma*	108	40	749861	Histoplasma	Unadjusted
1988	*Histoplasma*	111	43	924401	Histoplasma	Unadjusted
1989	*Histoplasma*	146	56	1078485	Histoplasma	Unadjusted
1990	*Histoplasma*	186	57	1698140	Histoplasma	Unadjusted
1991	*Histoplasma*	256	72	1008084	Histoplasma	Unadjusted
1992	*Histoplasma*	230	70	684471	Histoplasma	Unadjusted
1993	*Histoplasma*	243	68	1182692	Histoplasma	Unadjusted
1994	*Histoplasma*	250	70	1216820	Histoplasma	Unadjusted
1995	*Histoplasma*	292	75	1135682	Histoplasma	Unadjusted
1996	*Histoplasma*	245	66	781395	Histoplasma	Unadjusted
1997	*Histoplasma*	294	70	1908270	Histoplasma	Unadjusted
1998	*Histoplasma*	321	88	1702767	Histoplasma	Unadjusted
1999	*Histoplasma*	312	88	3355261	Histoplasma	Unadjusted
2000	*Histoplasma*	324	81	69513036	Histoplasma	Unadjusted
2001	*Histoplasma*	285	75	73246791	Histoplasma	Unadjusted
2002	*Histoplasma*	247	62	81239782	Histoplasma	Unadjusted
2003	*Histoplasma*	320	66	75243202	Histoplasma	Unadjusted
2004	*Histoplasma*	280	62	92705882	Histoplasma	Unadjusted
2005	*Histoplasma*	402	87	85957647	Histoplasma	Unadjusted
2006	*Histoplasma*	303	78	59152881	Histoplasma	Unadjusted
2007	*Histoplasma*	386	94	3246547	Histoplasma	Unadjusted
2008	*Histoplasma*	381	90	3493015	Histoplasma	Unadjusted
2009	*Histoplasma*	378	85	4012954	Histoplasma	Unadjusted
2010	*Histoplasma*	477	98	3057359	Histoplasma	Unadjusted
2011	*Histoplasma*	558	113	5628461	Histoplasma	Unadjusted
2012	*Histoplasma*	493	107	4969874	Histoplasma	Unadjusted
2013	*Histoplasma*	589	112	4996317	Histoplasma	Unadjusted
2014	*Histoplasma*	540	103	4572462	Histoplasma	Unadjusted
2015	*Histoplasma*	494	95	4435976	Histoplasma	Unadjusted
2016	*Histoplasma*	586	116	4398259	Histoplasma	Unadjusted
2017	*Histoplasma*	634	118	3358569	Histoplasma	Unadjusted
2018	*Histoplasma*	802	128	4425799	Histoplasma	Unadjusted
2019	*Histoplasma*	633	121	6730241	Histoplasma	Unadjusted
2020	*Histoplasma*	704	138	8436811	Histoplasma	Unadjusted
2021	*Histoplasma*	842	139	9750180	Histoplasma	Unadjusted
2022	*Histoplasma*	669	116	11182017	Histoplasma	Unadjusted
1985	*Blastomyces*	60	22	189181	Blastomyces	Unadjusted
1986	*Blastomyces*	79	27	326233	Blastomyces	Unadjusted
1987	*Blastomyces*	52	21	334465	Blastomyces	Unadjusted
1988	*Blastomyces*	49	22	390634	Blastomyces	Unadjusted
1989	*Blastomyces*	56	23	64285	Blastomyces	Unadjusted
1990	*Blastomyces*	71	28	185755	Blastomyces	Unadjusted
1991	*Blastomyces*	47	18	332282	Blastomyces	Unadjusted
1992	*Blastomyces*	102	26	344442	Blastomyces	Unadjusted
1993	*Blastomyces*	82	24	290101	Blastomyces	Unadjusted
1994	*Blastomyces*	96	27	153682	Blastomyces	Unadjusted
1995	*Blastomyces*	79	26	232774	Blastomyces	Unadjusted
1997	*Blastomyces*	75	26	473919	Blastomyces	Unadjusted
1998	*Blastomyces*	95	29	531294	Blastomyces	Unadjusted
1999	*Blastomyces*	66	26	585749	Blastomyces	Unadjusted
2000	*Blastomyces*	114	34	707047	Blastomyces	Unadjusted
2001	*Blastomyces*	78	23	745965	Blastomyces	Unadjusted
2002	*Blastomyces*	86	29	1391258	Blastomyces	Unadjusted
2003	*Blastomyces*	83	19	1317354	Blastomyces	Unadjusted
2004	*Blastomyces*	71	19	2102540	Blastomyces	Unadjusted
2005	*Blastomyces*	97	26	2164646	Blastomyces	Unadjusted
2006	*Blastomyces*	146	35	1785409	Blastomyces	Unadjusted
2007	*Blastomyces*	99	25	1315705	Blastomyces	Unadjusted
2008	*Blastomyces*	92	21	1486943	Blastomyces	Unadjusted
2009	*Blastomyces*	70	18	1838560	Blastomyces	Unadjusted
2010	*Blastomyces*	122	25	1562741	Blastomyces	Unadjusted
2011	*Blastomyces*	168	36	3669200	Blastomyces	Unadjusted
2012	*Blastomyces*	133	27	2788300	Blastomyces	Unadjusted
2013	*Blastomyces*	159	35	2145993	Blastomyces	Unadjusted
2014	*Blastomyces*	138	28	1935317	Blastomyces	Unadjusted
2015	*Blastomyces*	198	37	2540350	Blastomyces	Unadjusted
2016	*Blastomyces*	172	35	848351	Blastomyces	Unadjusted
2017	*Blastomyces*	167	39	1364204	Blastomyces	Unadjusted
2018	*Blastomyces*	139	36	2328193	Blastomyces	Unadjusted
2019	*Blastomyces*	143	34	2604316	Blastomyces	Unadjusted
2020	*Blastomyces*	222	52	2571734	Blastomyces	Unadjusted
2021	*Blastomyces*	226	44	2247852	Blastomyces	Unadjusted
2022	*Blastomyces*	200	34	3445199	Blastomyces	Unadjusted
1985	*Coccidioides*	91	32	402195	Coccidioides	Unadjusted
1986	*Coccidioides*	82	30	211368	Coccidioides	Unadjusted
1987	*Coccidioides*	64	24	399922	Coccidioides	Unadjusted
1988	*Coccidioides*	85	34	376952	Coccidioides	Unadjusted
1989	*Coccidioides*	66	23	638012	Coccidioides	Unadjusted
1990	*Coccidioides*	96	35	605801	Coccidioides	Unadjusted
1991	*Coccidioides*	72	26	626703	Coccidioides	Unadjusted
1992	*Coccidioides*	115	36	603085	Coccidioides	Unadjusted
1993	*Coccidioides*	116	37	681964	Coccidioides	Unadjusted
1994	*Coccidioides*	80	24	678134	Coccidioides	Unadjusted
1995	*Coccidioides*	95	31	1100479	Coccidioides	Unadjusted
1996	*Coccidioides*	107	34	654925	Coccidioides	Unadjusted
1997	*Coccidioides*	101	32	2067330	Coccidioides	Unadjusted
1998	*Coccidioides*	158	38	1251290	Coccidioides	Unadjusted
1999	*Coccidioides*	125	42	2447259	Coccidioides	Unadjusted
2000	*Coccidioides*	180	44	2638969	Coccidioides	Unadjusted
2001	*Coccidioides*	152	45	2572578	Coccidioides	Unadjusted
2002	*Coccidioides*	131	35	2420248	Coccidioides	Unadjusted
2003	*Coccidioides*	128	40	1182909	Coccidioides	Unadjusted
2004	*Coccidioides*	88	25	476940	Coccidioides	Unadjusted
2005	*Coccidioides*	194	55	472205	Coccidioides	Unadjusted
2006	*Coccidioides*	219	60	431696	Coccidioides	Unadjusted
2007	*Coccidioides*	181	65	1655403	Coccidioides	Unadjusted
2008	*Coccidioides*	192	40	1997152	Coccidioides	Unadjusted
2009	*Coccidioides*	196	43	2704194	Coccidioides	Unadjusted
2010	*Coccidioides*	248	52	3085004	Coccidioides	Unadjusted
2011	*Coccidioides*	311	58	2154585	Coccidioides	Unadjusted
2012	*Coccidioides*	240	48	2220705	Coccidioides	Unadjusted
2013	*Coccidioides*	311	58	2072489	Coccidioides	Unadjusted
2014	*Coccidioides*	277	57	2409621	Coccidioides	Unadjusted
2015	*Coccidioides*	311	64	4046782	Coccidioides	Unadjusted
2016	*Coccidioides*	267	59	4159837	Coccidioides	Unadjusted
2017	*Coccidioides*	292	62	5202984	Coccidioides	Unadjusted
2018	*Coccidioides*	355	66	5290889	Coccidioides	Unadjusted
2019	*Coccidioides*	390	82	15066973	Coccidioides	Unadjusted
2020	*Coccidioides*	355	79	8982218	Coccidioides	Unadjusted
2021	*Coccidioides*	425	83	7535550	Coccidioides	Unadjusted
2022	*Coccidioides*	394	71	15151439	Coccidioides	Unadjusted
1985	*Aspergillus*	1,116	370	2071242	Aspergillus	Unadjusted
1986	*Aspergillus*	1,087	353	1948595	Aspergillus	Unadjusted
1987	*Aspergillus*	1,209	409	3406249	Aspergillus	Unadjusted
1988	*Aspergillus*	1,275	435	3553480	Aspergillus	Unadjusted
1989	*Aspergillus*	1,588	502	4716809	Aspergillus	Unadjusted
1990	*Aspergillus*	1,749	555	4164373	Aspergillus	Unadjusted
1991	*Aspergillus*	1,783	534	5630289	Aspergillus	Unadjusted
1992	*Aspergillus*	2,034	600	4227152	Aspergillus	Unadjusted
1993	*Aspergillus*	2,164	663	4067380	Aspergillus	Unadjusted
1994	*Aspergillus*	2,427	725	3171370	Aspergillus	Unadjusted
1995	*Aspergillus*	2,590	752	2647943	Aspergillus	Unadjusted
1996	*Aspergillus*	2,740	745	975193	Aspergillus	Unadjusted
1997	*Aspergillus*	2,990	818	4700739	Aspergillus	Unadjusted
1998	*Aspergillus*	3,193	852	5338489	Aspergillus	Unadjusted
1999	*Aspergillus*	3,359	904	7433625	Aspergillus	Unadjusted
2000	*Aspergillus*	3,985	999	8629191	Aspergillus	Unadjusted
2001	*Aspergillus*	3,791	985	6786490	Aspergillus	Unadjusted
2002	*Aspergillus*	4,212	1,094	5844027	Aspergillus	Unadjusted
2003	*Aspergillus*	4,236	1,079	8042921	Aspergillus	Unadjusted
2004	*Aspergillus*	4,815	1,200	8037141	Aspergillus	Unadjusted
2005	*Aspergillus*	5,079	1,291	8299556	Aspergillus	Unadjusted
2006	*Aspergillus*	5,193	1,281	11631900	Aspergillus	Unadjusted
2007	*Aspergillus*	5,749	1,356	25058784	Aspergillus	Unadjusted
2008	*Aspergillus*	6,169	1,461	27333394	Aspergillus	Unadjusted
2009	*Aspergillus*	6,217	1,482	32002786	Aspergillus	Unadjusted
2010	*Aspergillus*	6,844	1,590	29589088	Aspergillus	Unadjusted
2011	*Aspergillus*	7,593	1,782	24982918	Aspergillus	Unadjusted
2012	*Aspergillus*	8,203	1,912	27833361	Aspergillus	Unadjusted
2013	*Aspergillus*	8,450	1,866	29266099	Aspergillus	Unadjusted
2014	*Aspergillus*	8,855	1,988	28678917	Aspergillus	Unadjusted
2015	*Aspergillus*	9,470	2,043	31926003	Aspergillus	Unadjusted
2016	*Aspergillus*	9,351	2,013	32916884	Aspergillus	Unadjusted
2017	*Aspergillus*	9,864	2,072	37912410	Aspergillus	Unadjusted
2018	*Aspergillus*	10,928	2,264	41729508	Aspergillus	Unadjusted
2019	*Aspergillus*	11,097	2,318	44498117	Aspergillus	Unadjusted
2020	*Aspergillus*	12,674	2,627	42612503	Aspergillus	Unadjusted
2021	*Aspergillus*	13,808	2,851	45086496	Aspergillus	Unadjusted
2022	*Aspergillus*	13,602	2,835	35282327	Aspergillus	Adjusted
1985	*Cryptococcus*	287	104	2083159.08	Cryptococcus	Adjusted
1986	*Cryptococcus*	281	96	2985597.43	Cryptococcus	Adjusted
1987	*Cryptococcus*	320	95	4645574.99	Cryptococcus	Adjusted
1988	*Cryptococcus*	369	111	3860116.56	Cryptococcus	Adjusted
1989	*Cryptococcus*	488	141	3816717.94	Cryptococcus	Adjusted
1990	*Cryptococcus*	494	142	5560049.21	Cryptococcus	Adjusted
1991	*Cryptococcus*	580	174	10396937	Cryptococcus	Adjusted
1992	*Cryptococcus*	572	168	12386658.6	Cryptococcus	Adjusted
1993	*Cryptococcus*	650	187	12371682.2	Cryptococcus	Adjusted
1994	*Cryptococcus*	674	208	10570817.4	Cryptococcus	Adjusted
1995	*Cryptococcus*	747	218	11376848.5	Cryptococcus	Adjusted
1996	*Cryptococcus*	825	229	2498467.16	Cryptococcus	Adjusted
1997	*Cryptococcus*	891	253	17159176.5	Cryptococcus	Adjusted
1998	*Cryptococcus*	954	257	16787932.4	Cryptococcus	Adjusted
1999	*Cryptococcus*	979	268	20159126.6	Cryptococcus	Adjusted
2000	*Cryptococcus*	1,237	305	22231301.4	Cryptococcus	Adjusted
2001	*Cryptococcus*	1,108	278	22773002	Cryptococcus	Adjusted
2002	*Cryptococcus*	1,142	279	26759598.5	Cryptococcus	Adjusted
2003	*Cryptococcus*	1,025	274	24157852.8	Cryptococcus	Adjusted
2004	*Cryptococcus*	1,201	298	21473090.3	Cryptococcus	Adjusted
2005	*Cryptococcus*	1,345	346	20830452.1	Cryptococcus	Adjusted
2006	*Cryptococcus*	1,411	357	17444523.1	Cryptococcus	Adjusted
2007	*Cryptococcus*	1,411	331	22256870.1	Cryptococcus	Adjusted
2008	*Cryptococcus*	1,544	362	22977371.7	Cryptococcus	Adjusted
2009	*Cryptococcus*	1,608	371	41225515.5	Cryptococcus	Adjusted
2010	*Cryptococcus*	1,914	426	43403509.4	Cryptococcus	Adjusted
2011	*Cryptococcus*	2,010	434	41994047.2	Cryptococcus	Adjusted
2012	*Cryptococcus*	2,185	473	36616069.2	Cryptococcus	Adjusted
2013	*Cryptococcus*	2,092	420	35245065.1	Cryptococcus	Adjusted
2014	*Cryptococcus*	2,280	460	35004753.3	Cryptococcus	Adjusted
2015	*Cryptococcus*	2,621	514	47512463.2	Cryptococcus	Adjusted
2016	*Cryptococcus*	2,510	457	72372485.7	Cryptococcus	Adjusted
2017	*Cryptococcus*	2,509	474	73657413.4	Cryptococcus	Adjusted
2018	*Cryptococcus*	2,719	515	52909133	Cryptococcus	Adjusted
2019	*Cryptococcus*	3,096	563	55770449.8	Cryptococcus	Adjusted
2020	*Cryptococcus*	3,344	645	63361527.9	Cryptococcus	Adjusted
2021	*Cryptococcus*	3,290	571	59031970.2	Cryptococcus	Adjusted
2022	*Cryptococcus*	3,070	552	37044435	Cryptococcus	Adjusted
1985	*Candida*	2,197	728	12970257.1	Candida	Adjusted
1986	*Candida*	2,126	712	15146243.4	Candida	Adjusted
1987	*Candida*	2,308	748	20809490.4	Candida	Adjusted
1988	*Candida*	2,499	789	19892901.7	Candida	Adjusted
1989	*Candida*	3,023	911	19638227.4	Candida	Adjusted
1990	*Candida*	2,891	919	19259253.8	Candida	Adjusted
1991	*Candida*	3,133	951	22381580.6	Candida	Adjusted
1992	*Candida*	3,505	1,014	26028214.6	Candida	Adjusted
1993	*Candida*	3,512	1,008	24735463.6	Candida	Adjusted
1994	*Candida*	3,620	1,034	23266302.9	Candida	Adjusted
1995	*Candida*	3,896	1,128	23555554.4	Candida	Adjusted
1996	*Candida*	4,198	1,121	9041035.26	Candida	Adjusted
1997	*Candida*	4,221	1,138	29963698.2	Candida	Adjusted
1998	*Candida*	4,545	1,245	36725524	Candida	Adjusted
1999	*Candida*	4,674	1,269	46531255.9	Candida	Adjusted
2000	*Candida*	5,269	1,358	47810182.2	Candida	Adjusted
2001	*Candida*	5,691	1,506	49042257.5	Candida	Adjusted
2002	*Candida*	5,781	1,522	53346584.3	Candida	Adjusted
2003	*Candida*	5,973	1,494	49149206.4	Candida	Adjusted
2004	*Candida*	6,570	1,626	53833920.1	Candida	Adjusted
2005	*Candida*	6,687	1,674	47821374.3	Candida	Adjusted
2006	*Candida*	7,071	1,748	42943447.5	Candida	Adjusted
2007	*Candida*	7,587	1,747	80085309.6	Candida	Adjusted
2008	*Candida*	8,022	1,902	74535359.3	Candida	Adjusted
2009	*Candida*	8,566	2,010	99999520.5	Candida	Adjusted
2010	*Candida*	9,283	2,163	93063549.4	Candida	Adjusted
2011	*Candida*	10,185	2,337	89273640	Candida	Adjusted
2012	*Candida*	10,810	2,420	74736101.9	Candida	Adjusted
2013	*Candida*	11,394	2,446	63341990.8	Candida	Adjusted
2014	*Candida*	12,195	2,600	75924766.2	Candida	Adjusted
2015	*Candida*	13,047	2,726	84620042.9	Candida	Adjusted
2016	*Candida*	14,764	2,789	82945901	Candida	Adjusted
2017	*Candida*	13,627	2,753	90952070.5	Candida	Adjusted
2018	*Candida*	14,720	2,927	89146232.3	Candida	Adjusted
2019	*Candida*	15,651	3,085	107676942	Candida	Adjusted
2020	*Candida*	17,068	3,391	114371298	Candida	Adjusted
2021	*Candida*	18,517	3,560	107584003	Candida	Adjusted
2022	*Candida*	18,949	3,633	76864438	Candida	Adjusted
1985	*Histoplasma*	134	40	517610.468	Histoplasma	Adjusted
1986	*Histoplasma*	170	54	1002746.28	Histoplasma	Adjusted
1987	*Histoplasma*	108	40	1943301.75	Histoplasma	Adjusted
1988	*Histoplasma*	111	43	2300453.55	Histoplasma	Adjusted
1989	*Histoplasma*	146	56	2560532.13	Histoplasma	Adjusted
1990	*Histoplasma*	186	57	3825037.61	Histoplasma	Adjusted
1991	*Histoplasma*	256	72	2179000.95	Histoplasma	Adjusted
1992	*Histoplasma*	230	70	1436267.02	Histoplasma	Adjusted
1993	*Histoplasma*	243	68	2409581.49	Histoplasma	Adjusted
1994	*Histoplasma*	250	70	2417218.68	Histoplasma	Adjusted
1995	*Histoplasma*	292	75	2193863.39	Histoplasma	Adjusted
1996	*Histoplasma*	245	66	1466173.92	Histoplasma	Adjusted
1997	*Histoplasma*	294	70	3500278.43	Histoplasma	Adjusted
1998	*Histoplasma*	321	88	3075427.02	Histoplasma	Adjusted
1999	*Histoplasma*	312	88	5929104.67	Histoplasma	Adjusted
2000	*Histoplasma*	324	81	118842264	Histoplasma	Adjusted
2001	*Histoplasma*	285	75	121760899	Histoplasma	Adjusted
2002	*Histoplasma*	247	62	132946036	Histoplasma	Adjusted
2003	*Histoplasma*	320	66	120389123	Histoplasma	Adjusted
2004	*Histoplasma*	280	62	144481798	Histoplasma	Adjusted
2005	*Histoplasma*	402	87	129574661	Histoplasma	Adjusted
2006	*Histoplasma*	303	78	86381985	Histoplasma	Adjusted
2007	*Histoplasma*	386	94	4610629.22	Histoplasma	Adjusted
2008	*Histoplasma*	381	90	4776328.92	Histoplasma	Adjusted
2009	*Histoplasma*	378	85	5507755.98	Histoplasma	Adjusted
2010	*Histoplasma*	477	98	4126944.02	Histoplasma	Adjusted
2011	*Histoplasma*	558	113	7367803.11	Histoplasma	Adjusted
2012	*Histoplasma*	493	107	6372521.37	Histoplasma	Adjusted
2013	*Histoplasma*	589	112	6312943.03	Histoplasma	Adjusted
2014	*Histoplasma*	540	103	5687084.13	Histoplasma	Adjusted
2015	*Histoplasma*	494	95	5510343.18	Histoplasma	Adjusted
2016	*Histoplasma*	586	116	5395197.71	Histoplasma	Adjusted
2017	*Histoplasma*	634	118	4034119.6	Histoplasma	Adjusted
2018	*Histoplasma*	802	128	5188989.35	Histoplasma	Adjusted
2019	*Histoplasma*	633	121	7748857.84	Histoplasma	Adjusted
2020	*Histoplasma*	704	138	9597361.51	Histoplasma	Adjusted
2021	*Histoplasma*	842	139	10592077.5	Histoplasma	Adjusted
2022	*Histoplasma*	669	116	11182017	Histoplasma	Adjusted
1985	*Blastomyces*	60	22	517610.468	Blastomyces	Adjusted
1986	*Blastomyces*	79	27	876304.701	Blastomyces	Adjusted
1987	*Blastomyces*	52	21	866782.535	Blastomyces	Adjusted
1988	*Blastomyces*	49	22	972127.216	Blastomyces	Adjusted
1989	*Blastomyces*	56	23	152625.032	Blastomyces	Adjusted
1990	*Blastomyces*	71	28	418410.65	Blastomyces	Adjusted
1991	*Blastomyces*	47	18	718236.57	Blastomyces	Adjusted
1992	*Blastomyces*	102	26	722763.541	Blastomyces	Adjusted
1993	*Blastomyces*	82	24	591043.145	Blastomyces	Adjusted
1994	*Blastomyces*	96	27	305290.019	Blastomyces	Adjusted
1995	*Blastomyces*	79	26	449663.16	Blastomyces	Adjusted
1997	*Blastomyces*	75	26	869294.415	Blastomyces	Adjusted
1998	*Blastomyces*	95	29	959588.672	Blastomyces	Adjusted
1999	*Blastomyces*	66	26	1035081.07	Blastomyces	Adjusted
2000	*Blastomyces*	114	34	1208795.8	Blastomyces	Adjusted
2001	*Blastomyces*	78	23	1240045.71	Blastomyces	Adjusted
2002	*Blastomyces*	86	29	2276744.61	Blastomyces	Adjusted
2003	*Blastomyces*	83	19	2107766.4	Blastomyces	Adjusted
2004	*Blastomyces*	71	19	3276801.36	Blastomyces	Adjusted
2005	*Blastomyces*	97	26	3263040.36	Blastomyces	Adjusted
2006	*Blastomyces*	146	35	2607263.94	Blastomyces	Adjusted
2007	*Blastomyces*	99	25	1868516.89	Blastomyces	Adjusted
2008	*Blastomyces*	92	21	2033237.43	Blastomyces	Adjusted
2009	*Blastomyces*	70	18	2523412.89	Blastomyces	Adjusted
2010	*Blastomyces*	122	25	2109449.57	Blastomyces	Adjusted
2011	*Blastomyces*	168	36	4803079.06	Blastomyces	Adjusted
2012	*Blastomyces*	133	27	3575241.81	Blastomyces	Adjusted
2013	*Blastomyces*	159	35	2711503.6	Blastomyces	Adjusted
2014	*Blastomyces*	138	28	2407086.29	Blastomyces	Adjusted
2015	*Blastomyces*	198	37	3155607.76	Blastomyces	Adjusted
2016	*Blastomyces*	172	35	1040643.89	Blastomyces	Adjusted
2017	*Blastomyces*	167	39	1638603.25	Blastomyces	Adjusted
2018	*Blastomyces*	139	36	2729669.53	Blastomyces	Adjusted
2019	*Blastomyces*	143	34	2998477.24	Blastomyces	Adjusted
2020	*Blastomyces*	222	52	2925496.48	Blastomyces	Adjusted
2021	*Blastomyces*	226	44	2441946.97	Blastomyces	Adjusted
2022	*Blastomyces*	200	34	3445199	Blastomyces	Adjusted
1985	*Coccidioides*	91	32	1100429.44	Coccidioides	Adjusted
1986	*Coccidioides*	82	30	567762.219	Coccidioides	Adjusted
1987	*Coccidioides*	64	24	1036417.58	Coccidioides	Adjusted
1988	*Coccidioides*	85	34	938078.35	Coccidioides	Adjusted
1989	*Coccidioides*	66	23	1514763.97	Coccidioides	Adjusted
1990	*Coccidioides*	96	35	1364558.64	Coccidioides	Adjusted
1991	*Coccidioides*	72	26	1354635.56	Coccidioides	Adjusted
1992	*Coccidioides*	115	36	1265489.84	Coccidioides	Adjusted
1993	*Coccidioides*	116	37	1389413.16	Coccidioides	Adjusted
1994	*Coccidioides*	80	24	1347116.39	Coccidioides	Adjusted
1995	*Coccidioides*	95	31	2125859.7	Coccidioides	Adjusted
1996	*Coccidioides*	107	34	1228871.38	Coccidioides	Adjusted
1997	*Coccidioides*	101	32	3792037.08	Coccidioides	Adjusted
1998	*Coccidioides*	158	38	2259998.63	Coccidioides	Adjusted
1999	*Coccidioides*	125	42	4324568.13	Coccidioides	Adjusted
2000	*Coccidioides*	180	44	4511686.84	Coccidioides	Adjusted
2001	*Coccidioides*	152	45	4276493.3	Coccidioides	Adjusted
2002	*Coccidioides*	131	35	3960650.42	Coccidioides	Adjusted
2003	*Coccidioides*	128	40	1892654.4	Coccidioides	Adjusted
2004	*Coccidioides*	88	25	743309.349	Coccidioides	Adjusted
2005	*Coccidioides*	194	55	711813.374	Coccidioides	Adjusted
2006	*Coccidioides*	219	60	630413.206	Coccidioides	Adjusted
2007	*Coccidioides*	181	65	2350943.77	Coccidioides	Adjusted
2008	*Coccidioides*	192	40	2730894.33	Coccidioides	Adjusted
2009	*Coccidioides*	196	43	3711490.51	Coccidioides	Adjusted
2010	*Coccidioides*	248	52	4164260.33	Coccidioides	Adjusted
2011	*Coccidioides*	311	58	2820408.29	Coccidioides	Adjusted
2012	*Coccidioides*	240	48	2847454.5	Coccidioides	Adjusted
2013	*Coccidioides*	311	58	2618629.88	Coccidioides	Adjusted
2014	*Coccidioides*	277	57	2997010.66	Coccidioides	Adjusted
2015	*Coccidioides*	311	64	5026888.7	Coccidioides	Adjusted
2016	*Coccidioides*	267	59	5102733.39	Coccidioides	Adjusted
2017	*Coccidioides*	292	62	6249524.64	Coccidioides	Adjusted
2018	*Coccidioides*	355	66	6203256.56	Coccidioides	Adjusted
2019	*Coccidioides*	390	82	17347347.9	Coccidioides	Adjusted
2020	*Coccidioides*	355	79	10217793.6	Coccidioides	Adjusted
2021	*Coccidioides*	425	83	8186221.11	Coccidioides	Adjusted
2022	*Coccidioides*	394	71	15151439	Coccidioides	Adjusted
1985	*Aspergillus*	1,116	370	5667041.31	Aspergillus	Adjusted
1986	*Aspergillus*	1,087	353	5234182.19	Aspergillus	Adjusted
1987	*Aspergillus*	1,209	409	8827462.2	Aspergillus	Adjusted
1988	*Aspergillus*	1,275	435	8843148.88	Aspergillus	Adjusted
1989	*Aspergillus*	1,588	502	11198617.5	Aspergillus	Adjusted
1990	*Aspergillus*	1,749	555	9380194.42	Aspergillus	Adjusted
1991	*Aspergillus*	1,783	534	12170022.6	Aspergillus	Adjusted
1992	*Aspergillus*	2,034	600	8870089.44	Aspergillus	Adjusted
1993	*Aspergillus*	2,164	663	8286758.98	Aspergillus	Adjusted
1994	*Aspergillus*	2,427	725	6299941.48	Aspergillus	Adjusted
1995	*Aspergillus*	2,590	752	5115186.48	Aspergillus	Adjusted
1996	*Aspergillus*	2,740	745	1829807.64	Aspergillus	Adjusted
1997	*Aspergillus*	2,990	818	8622414.71	Aspergillus	Adjusted
1998	*Aspergillus*	3,193	852	9642031.67	Aspergillus	Adjusted
1999	*Aspergillus*	3,359	904	13136009.6	Aspergillus	Adjusted
2000	*Aspergillus*	3,985	999	14752809.7	Aspergillus	Adjusted
2001	*Aspergillus*	3,791	985	11281437.9	Aspergillus	Adjusted
2002	*Aspergillus*	4,212	1,094	9563543.91	Aspergillus	Adjusted
2003	*Aspergillus*	4,236	1,079	12868673.6	Aspergillus	Adjusted
2004	*Aspergillus*	4,815	1,200	12525856.6	Aspergillus	Adjusted
2005	*Aspergillus*	5,079	1,291	12510953.9	Aspergillus	Adjusted
2006	*Aspergillus*	5,193	1,281	16986266.7	Aspergillus	Adjusted
2007	*Aspergillus*	5,749	1,356	35587583.3	Aspergillus	Adjusted
2008	*Aspergillus*	6,169	1,461	37375528.1	Aspergillus	Adjusted
2009	*Aspergillus*	6,217	1,482	43923637.3	Aspergillus	Adjusted
2010	*Aspergillus*	6,844	1,590	39940520.4	Aspergillus	Adjusted
2011	*Aspergillus*	7,593	1,782	32703295.1	Aspergillus	Adjusted
2012	*Aspergillus*	8,203	1,912	35688769.5	Aspergillus	Adjusted
2013	*Aspergillus*	8,450	1,866	36978281.3	Aspergillus	Adjusted
2014	*Aspergillus*	8,855	1,988	35669933.1	Aspergillus	Adjusted
2015	*Aspergillus*	9,470	2,043	39658292.3	Aspergillus	Adjusted
2016	*Aspergillus*	9,351	2,013	40378044.4	Aspergillus	Adjusted
2017	*Aspergillus*	9,864	2,072	45538202.8	Aspergillus	Adjusted
2018	*Aspergillus*	10,928	2,264	48925396.9	Aspergillus	Adjusted
2019	*Aspergillus*	11,097	2,318	51232873.1	Aspergillus	Adjusted
2020	*Aspergillus*	12,674	2,627	48474192	Aspergillus	Adjusted
2021	*Aspergillus*	13,808	2,851	48979573.5	Aspergillus	Adjusted
2022	*Aspergillus*	13,602	2,835	35282327	Aspergillus	Adjusted
2018	*mucorales*	1,470	264	NA	Mucorales	NA
2018	*Pneumocystis*	1,520	266	NA	Pneumocystis	NA
2012	*Mucorales*	1,133	248	NA	Mucorales	NA
2012	*Pneumocystis*	1,338	237	NA	Pneumocystis	NA
2012	*Paracoccidioides*	417	77	NA	Paracoccidioides	NA
2012	*Penicillium marneffei*	179	27	NA	Penicillium+marneffei	NA
2018	Coronavirus	3,996	730	NA	Coronavirus	NA
2018	Influenza	23,586	5,151	NA	Influenza	NA
2018	Salmonellosis	6,651	1,162	NA	Salmonellosis	NA
2018	Zika virus	9,186	1,625	NA	Zika+virus	NA
2018	Viral hemorrhagic fevers	9,968	1,822	NA	Viral+hemorrhagic+fevers	NA
2018	Cholera	4,145	787	NA	Cholera	NA
2018	Dengue	10,015	1,912	NA	Dengue	NA
2018	Tuberculosis	33,864	7,667	NA	Tuberculosis	NA
2018	HIV	62,928	15,211	NA	Human+immunodeficiency+virus	NA
2018	Malaria	20,704	4,090	NA	Malaria	NA

Scientists mostly study pathogens and diseases of interest to them and for which resources are allocated, so we next investigated whether fields naturally stabilize to sizes reflecting the case burden of the disease. The WHO recently released a list of fungal pathogen priorities, and we compared the field size to the relative priority of each fungal disease. As expected, a larger proportion of the workforce is committed to critical priority pathogens, though the high-priority field is experiencing rapid growth (Fig. 3B and C).

To investigate more closely, we compared the size of fields working on various fungal diseases to the burden of disease caused by that fungus. Unfortunately, current reporting of fungal diseases is limited and not standardized. However, we were able to obtain reliable estimates of various fungal disease diagnoses for 2 years and compare the case burden to the size of fields. We found that, in both years, the number of authors reporting on a field had a slight positive correlation with case burden (Fig. 3D). Interestingly, when we compared funding per author to case burden, we found no strong correlation between the two (Fig. 3E).

**Fig 4:**
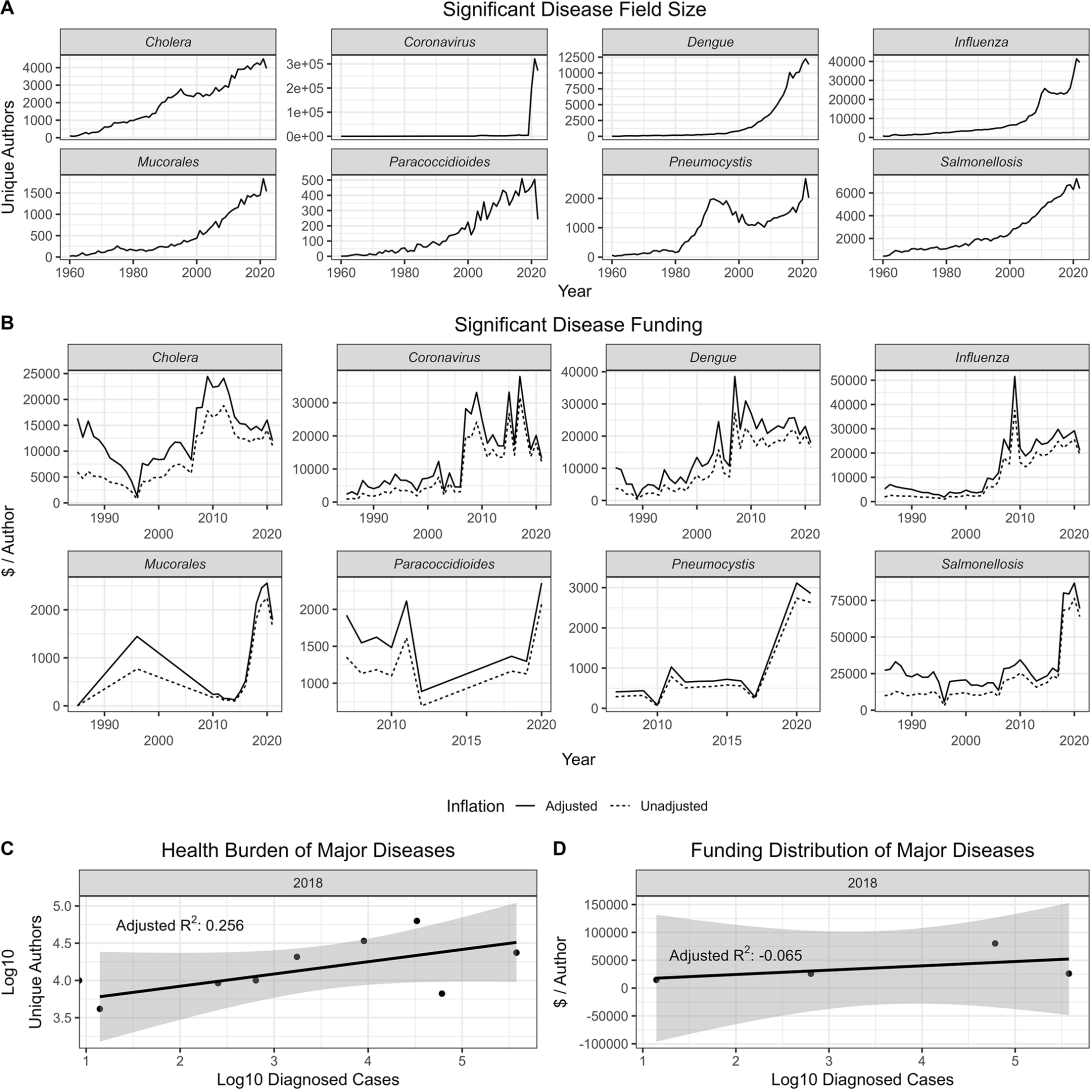
Size and funding of major human diseases. (A) Size of major human disease fields counted by unique authors. (B) Funding per author for significant human disease fields adjusted (solid) and unadjusted (dashed) for inflation. (C) Size of major disease fields compared to case burden in the US during 2018. (D) Funding per author compared to diagnosed case burden of significant human diseases. Adjusted *R*-squared values are shown for linear model regressions.

We next investigated whether these trends held true outside of fungal diseases by expanding our search to 10 major human diseases. Broadly, we observed similar results to the fungal field where the size of each field continually increased, funding per author was generally consistent, and neither field size nor funding correlated strongly with diagnosed case burden (Fig. 4). Several spikes in funding are noticeable after major events involving particular diseases: the 2018 Salmonellosis outbreak, the 2016 Zika outbreak, etc. For the most part, however, the workforce attributed to each category has been steadily increasing. The largest effects on the workforce, as one would expect, seem to follow diseases uncommon or previously unseen to the US breaking out (Zika outbreak in 2016, novel coronavirus in 2019, etc.).

### Unique authors mirror activity and use of model organisms and methods

**Fig 5:**
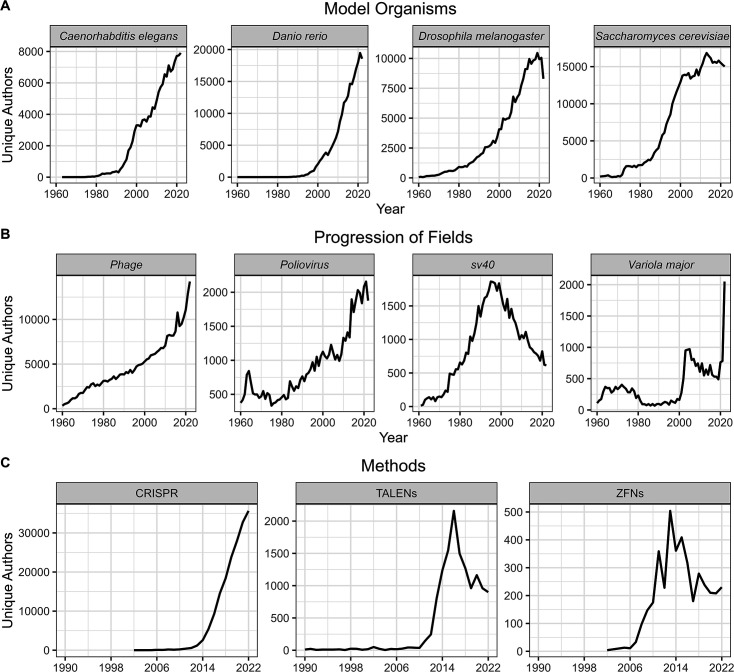
Growth and shrinking of fields. We analyzed four model organisms, four fields of research, and three iterations of gene editing technology to investigate how unique author count correlates to fields growing and changing over time. (A) We found that the size of model organism fields correlated well with their increased use. Interestingly, the *Drosophila* field eventually plateaued and was supplanted by the zebrafish. (B) We found that the authors in the SV40 field correlated well with its use in generating cancer models. Polio and smallpox mostly remained steady at lower numbers of unique authors per year. (C) Phage research experienced a steep increase in recent popularity. (D) TALENs, ZFNs, and CRISPR are three competing gene editing technologies, with clear field preferences. The popularity of each field is clearly visualized by author count with peaks and valleys expectedly following the development of each new technique.

One would expect the number of unique authors in a field to mirror a specific model organism or pathogen use, and outdated or early versions of methods are less likely to be used by new investigators. Thus, we analyzed several model organisms (*Saccharomyces cerevisiae, Danio rerio, Drosophila melanogaster,* and *Caenorhabditis elegans*), three pathogens we would not expect to continue increasing in size given advances in virology and vaccines (SV40, poliovirus, and *Variola major*), a field which experienced multiple popularity spikes from discoveries decades apart (phage), and three recent gene editing methods with overlapping userbases: zinc finger nucleases (ZFNs), transcription activator-like effector nucleases (TALENs), and clustered regularly interspaced short palindromic repeats (CRISPR). We observed expected patterns of field size according to use and prevalence over time for each category (Fig. 5).

**TABLE 4:** Statistics for linear regression models featured in Fig. 6A using linear and exponential growth rate models for sizes of fields

Query	Organism	Model	Search	Adj. *R*^2^	Sigma	Fstat
Cryptococcus	*Cryptococcus neoformans*	Linear	Genus	0.83	396.56	303.68
Cryptococcus+neoformans	*Cryptococcus neoformans*	Linear	Species	0.89	230.08	478.81
Candida	*Candida albicans*	Linear	Genus	0.82	2,156.39	286.66
Candida+albicans	*Candida albicans*	Linear	Species	0.84	1,286.37	324.04
Histoplasma	*Histoplasma capsulatum*	Linear	Genus	0.84	82.23	329.31
Histoplasma+capsulatum	*Histoplasma capsulatum*	Linear	Species	0.84	82.23	329.31
Blastomyces	*Blastomyces dermatitidis*	Linear	Genus	0.75	25.46	190.77
Blastomyces+dermatitidis	*Blastomyces dermatitidis*	Linear	Species	0.75	25.46	190.77
Coccidioides	*Coccidioides immitis*	Linear	Genus	0.79	49.26	230.86
Coccidioides+immitis	*Coccidioides immitis*	Linear	Species	0.78	48.06	218.67
Aspergillus	*Aspergillus fumigatus*	Linear	Genus	0.81	1,657.74	261.24
Aspergillus+fumigatus	*Aspergillus fumigatus*	Linear	Species	0.83	449.23	304.04
Cryptococcus	*Cryptococcus neoformans*	Exponential	Genus	0.96	0.23	1,614.16
Cryptococcus+neoformans	*Cryptococcus neoformans*	Exponential	Species	0.94	0.36	973.67
Candida	*Candida albicans*	Exponential	Genus	0.96	0.24	1,462.52
Candida+albicans	*Candida albicans*	Exponential	Species	0.94	0.37	922.9
Histoplasma	*Histoplasma capsulatum*	Exponential	Genus	0.93	0.21	870.86
Histoplasma+capsulatum	*Histoplasma capsulatum*	Exponential	Species	0.93	0.21	870.86
Blastomyces	*Blastomyces dermatitidis*	Exponential	Genus	0.74	0.36	174.36
Blastomyces+dermatitidis	*Blastomyces dermatitidis*	Exponential	Species	0.74	0.36	174.36
Coccidioides	*Coccidioides immitis*	Exponential	Genus	0.89	0.27	479.3
Coccidioides+immitis	*Coccidioides immitis*	Exponential	Species	0.88	0.28	461.84
Aspergillus	*Aspergillus fumigatus*	Exponential	Genus	0.96	0.24	1,596.76
Aspergillus+fumigatus	*Aspergillus fumigatus*	Exponential	Species	0.95	0.37	1,124.79

**Fig 6:**
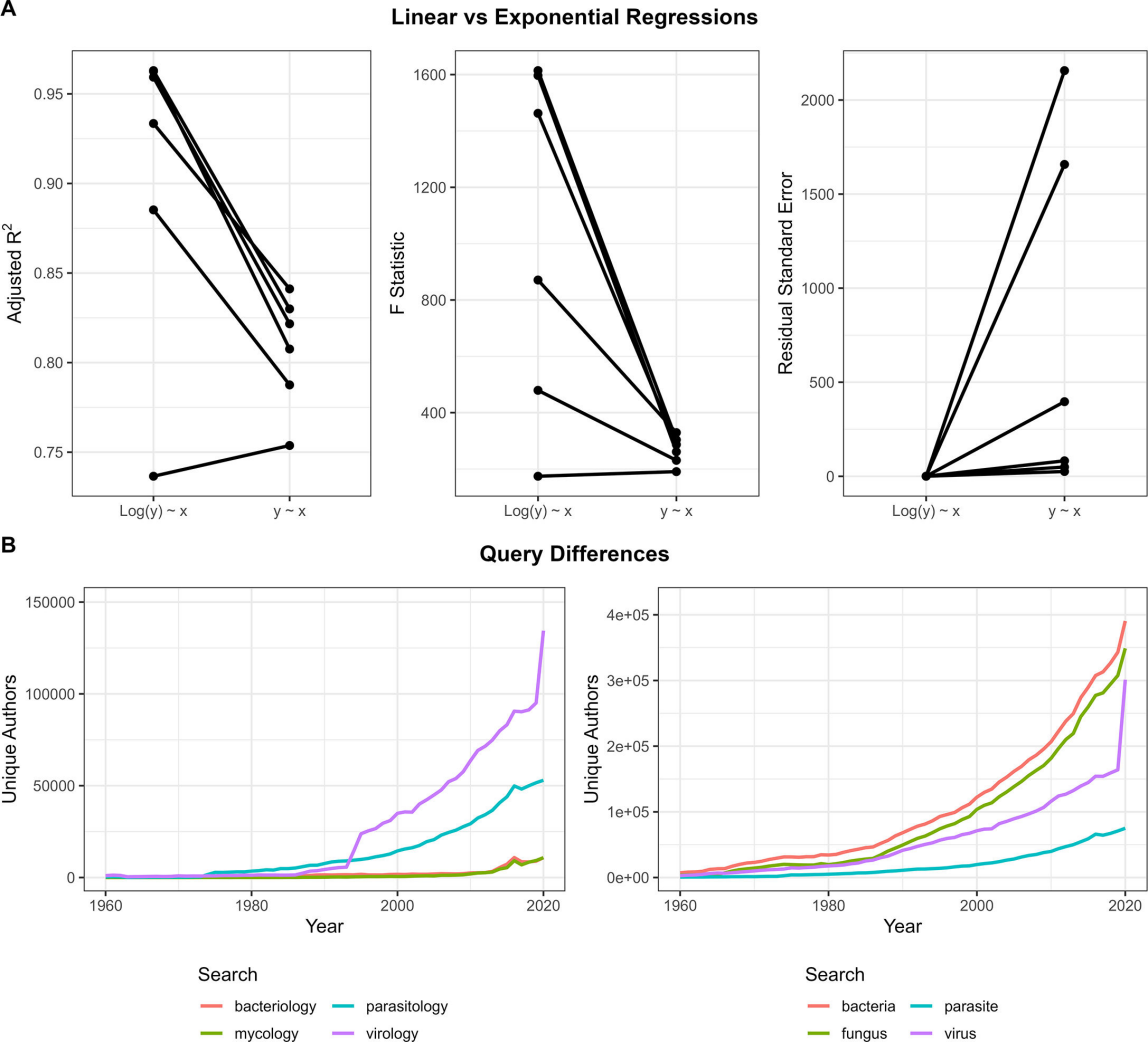
Growth analysis. (A) Comparisons of linear and exponential fits on growth rates of each scientific field analyzed in this paper. Linear regressions were performed on either the raw data or the log-transformed field sizes to compare the two models. Exponential regressions fit better overall, though clear exceptions exist. Detailed information included in Table 4. (B) Differences in results and comparisons based on differences in initial search queries. Terms like mycology vs fungus capture fewer articles, and care must be taken to craft a proper query.

## DISCUSSION

Understanding field size and organization is essential to effectively understand the structure of our scientific workforce. There is some evidence that scientists favor working on specific topics, which raises the concern that chasing “hot topics” could neglect essential basic science research (12). We hoped to develop a measurement of field size that could help quantify these differences and determine how and where our scientific work effort is distributed. To compile our analysis, we used Entrez Direct searches of the PubMed database for each query. These searches match queries to text in the title, abstract, and keywords of articles while ignoring text in the introduction, results, and discussions. Thus, it is unlikely that our results would be contaminated by the inclusion of search terms in background text and that searches return papers that are actually associated with a given query. The use of unique authors in a field differs from merely counting total publications in that it offers additional dimensions of analysis. Publication counts reflect the research output of a field as a whole, but knowing how that output is distributed across different laboratories and working environments gives a more granular image of the research landscape. Diversity of ideas and research groups is essential to creative science and fostering new ideas whereas fields dominated by fewer smaller lab groups may find themselves stymied from dogmatic hierarchies.

Measuring unique authors also avoids certain pitfalls associated with paper number-driven analyses. First, it allows us to control for differences in output between individual authors and fields. Since we are looking to measure effort and workforce rather than productivity, a single author who outputs 10 articles per year should be weighed the same as an author who outputs 15. Conversely, a paper with 10 authors implies a larger workforce behind it than a paper with 2. Thus, quantifying effort and personnel according to papers does not allow for granularity in terms of personnel and may explain why we sometimes observed differences in field size when comparing total paper counts to unique author counts.

We propose that counting unique authors provides an estimate of the size and activity of a given field. The overall number of papers published in a field each year trended strongly with the total number of unique authors across many, but not all, of the analyzed fields. We included searches of both genus and genus species to ensure the specificity of search terms, for example, determining whether searching “*Aspergillus fumigatus*” would return “*Aspergillus niger*” papers. We also compared searches of individual known authors and specific paper topics to ensure sub-selections of papers would be returned correctly. Across each test, PubMed returned the correct corpus of articles.

Once validated, we applied regression analyses to the size of each field over time to determine growth rates and dynamics. We found that exponential growth seemed to best describe the changing size of fields overall, though each field had their own unique patterns, and some have even begun to decline. Perpetual exponential growth is not infinitely maintainable, so we expect every field size to eventually plateau. However, it is interesting that just about every field experienced exponential growth, even those without heavily publicized outbreaks.

NIH RePORTER and CDC Required Reporting data are limited in the context of these diseases, but we were able to identify a slight trend of increasing funding per author over time. When comparing the size of a given field to the public health burden of its respective disease, we noticed a slight positive correlation across time and irrespective of whether the focus was on global cases or limited to the US. We observed a similar trend when comparing the WHO priority list of fungal pathogens, noting that critical fungal pathogens on average had a larger proportion of the workforce, followed by high priority, followed by medium priority. This analysis is reassuring in that the allocated labor force appears to be efficiently focusing on microbes with high disease burden while not neglecting microbes with lower-burden diseases. This trend persisted even when comparing fungal diseases to more globally significant human diseases such as HIV, malaria, and tuberculosis. These larger fields experienced similar growth over time, but the distribution of funding remained similar to the fungal fields, further reassuring us that resources and workforce distribute between fields of research rather than disproportionately flocking to perceived “hot topic” research.

Our analysis also provides insight into the growth and decline of fields over time. For example, the use and popularity of the *S. cerevisiae* system as a model for eukaryotic cell biology rose rapidly in the 1970s–1980s and then stabilized, presumably as mammalian cell systems matured to allow comparable types of experimentation. In 1990, the estimated size of the four model organisms was *S. cerevisiae* > *D. melanogaster* > *C. elegans,* while zebrafish had not yet emerged as a major model organism. Three decades later, there are more authors associated with zebrafish than the other model organisms, and *S. cerevisiae* has moved to second place. We interpret this sequence as reflecting the fact that for eukaryotic cell biology, molecular techniques for studying deep questions were available first in yeast, then invertebrate flies, and most recently in vertebrate zebrafish. In virology, SV40, a polyoma virus that can cause tumors, was a major early experimental system used to understand how viruses caused cancer, but its popularity has declined as investigators have moved to other systems. Poliovirus was a major medical problem before the introduction of effective vaccines in the 1950s, and the number of authors associated with papers on this virus remained relatively stable until recent years when the virus resurfaced with new strains and a decline in vaccination in some regions. Similarly, smallpox was a scourge in the past but was eradicated in 1977 and followed by a steady decline around 1990, then a resurgence of interest with concerns about its potential as a biological weapon and increased interest given human pathogenic potential of other poxviruses. Hence, for both model systems and three major viruses, the trends in author numbers can be associated with historical developments in their fields.

Our analysis has several caveats and limitations. First, there is no complete standardization of the author name format in PubMed, meaning that the same author may appear and be counted twice in our analysis if, for example, their name appears as last first and last first-initial on separate publications. We were able to identify and correct this issue for authors we personally knew, but it would be impossible to parse out initials from more common names (e.g., Smith and Nguyen). The increasing prevalence of ORCIDs may fix this problem in the future. We also caution that field size estimated from author numbers is likely to be an upper-ceiling estimate since not everyone who authors a paper in an area is necessarily doing research in the area in question. For example, the large increase in investigators authoring phage and CRISPR papers likely reflects the usefulness of those systems for a variety of high-throughput experiments, rather than an increase in the numbers of individuals working on phage biology or CRISPR machinery. Database selection is another important consideration. While PubMed is the *de facto* leader in medical microbiology, other fields wishing to use this system may require access to alternate databases. Finally, investigators must take care to properly format search queries as even very small changes can yield drastically different results. For example, there are several orders of magnitude differences in the size of “bacteria” compared to “bacteriology.” Finally, in considering the temporal changes in fields, we caution that the number of journals published and indexed in PubMed has increased over the years, which could have increased the number of unique authors.

In summary, we propose a bibliometric method for estimating the size of scientific fields and apply it to microbiology and several subfields. The availability of large open bibliometric databases combined with software tools for their analysis provides the means to study problems that were previously inaccessible, and there are many questions that can be explored using these approaches. We note that there is little scholarship on this topic, and our analysis should be considered a starting step in the exploration of a complex topic of great importance to humanity. When applied to the subfield of medical mycology, the results show differences in the size of fields that correlate with the medical importance of a particular fungus. We are hopeful that this approach provides a useful tool for sociologists of science and policymakers for studying the structure of scientific fields.
